# Case Report: Sequential Combination Targeted Therapy With Type I and II MET Inhibitors in a Metastatic *EGFR*-Mutated, *MET*-Amplified NSCLC Patient With Acquired *MET* Y1230H Mutation

**DOI:** 10.3389/fonc.2021.738832

**Published:** 2021-12-02

**Authors:** Boning Cai, Xiaomo Li, Xiang Huang, Tonghui Ma, Baolin Qu, Wei Yu, Wei Yang, Pei Zhang, Jing Chen, Fang Liu

**Affiliations:** ^1^ Department of Radiation Oncology, The First Medical Center, Chinese People’s Liberation Army (PLA) General Hospital, Beijing, China; ^2^ Department of Translational Medicine, Genetron Health (Beijing) Technology, Co. Ltd, Beijing, China

**Keywords:** non-small cell lung cancer (NSCLC), *EGFR* mutation, targeted therapy resistance, *MET* amplification, case report

## Abstract

Epidermal growth factor receptor (EGFR) tyrosine kinase inhibitors (TKIs) are the standard of care for advanced non-small-cell lung cancer (NSCLC) patients. However, most patients will eventually develop resistance. For EGFR-TKI resistance mediated by *MET* amplification, the combination of EGFR and MET TKIs has shown promising results in early clinical trials. However, acquired resistance to MET inhibitors forms a formidable challenge to this dual blockade approach. Here, we presented an NSCLC patient with *EGFR* exon 19 deletion (ex19del) who was resistant to first-line erlotinib treatment but responded to chemotherapy. Given the finding of *MET* overexpression/amplification after disease progression, the patient received gefitinib plus crizotinib with a partial response. Her disease progressed again, and molecular testing revealed a novel *MET* Y1230H mutation and a PD-L1 TPS score of 75%. She received a salvage regime consisting of gefitinib, cabozantinib, and pembrolizumab with a partial response. Since we now know that *EGFR* ex19del NSCLC patients generally do not respond to PD-1 blockade therapy, this response is more likely the contribution from gefitinib plus cabozantinib. Therefore, sequential use of type I and II MET inhibitors in EGFR/MET dual blockade may be an effective therapeutic option for *EGFR*-mutant, *MET*-amplified NSCLC.

## Introduction

Epidermal growth factor receptor (*EGFR*) mutations are present in ~30-50% of non-small cell lung cancer (NSCLC) cases in East Asia and ~10% of cases in North America and Western Europe (1). EGFR tyrosine kinase inhibitors (EGFR-TKIs) are the standard of care for advanced *EGFR*-mutated NSCLC (2). As most NSCLC patients initially treated with EGFR TKIs will eventually acquire resistance, strategies to overcome EGFR TKI resistance are needed to improve patient outcomes. *EGFR* T790M mutation and *MET* amplification are the dominant on-target and off-target EGFR TKI resistance mechanisms, respectively (3). While the third-generation EGFR TKI osimertinib can overcome resistance mediated by *EGFR* T790M, resistance mediated by *MET* amplification remains a challenge (4).

Genetic alterations of *MET* are new therapeutic targets in NSCLC (4, 5). Three MET TKIs (capmatinib, tepotinib, and savolitinib) have been approved as first-line treatment for NSCLC patients with *MET* exon 14 skipping (*MET*ex14) mutations (6–8). Based on the mechanism of action, MET TKIs are divided into two groups, type I and type II, which bind to the active and inactive form of the ATP-pocket of MET, respectively (9). However, some acquired *MET* mutations such as Y1230H result in resistance to all type I MET inhibitors approved for NSCLC (10, 11). Furthermore, there is no approved targeted therapy for NSCLC patients with *MET* amplification, a less frequent driver in NSCLC compared with *MET*ex14 mutations (4). Therefore, the clinical management of *MET*-amplified NSCLC and acquired MET TKI resistance represent two clinical challenges.

To overcome EGFR TKI resistance mediated by *MET* amplification, clinicians are testing different EGFR/MET TKI combinations in clinical trials (12–15). Additionally, off-label use of type II MET TKI cabozantinib can be a feasible strategy to overcome acquired MET TKI resistance in *MET*ex14-NSCLC (4). Here, we described a metastatic *EGFR*-mutant NSCLC patient who developed EGFR TKI resistance mediated by *MET* overexpression/amplification and subsequently responded to gefitinib plus crizotinib. This patient then developed resistance to crizotinib due to an acquired *MET* Y1230H mutation, which was overcome by cabozantinib.

## Case Presentation

**Figure 1:**
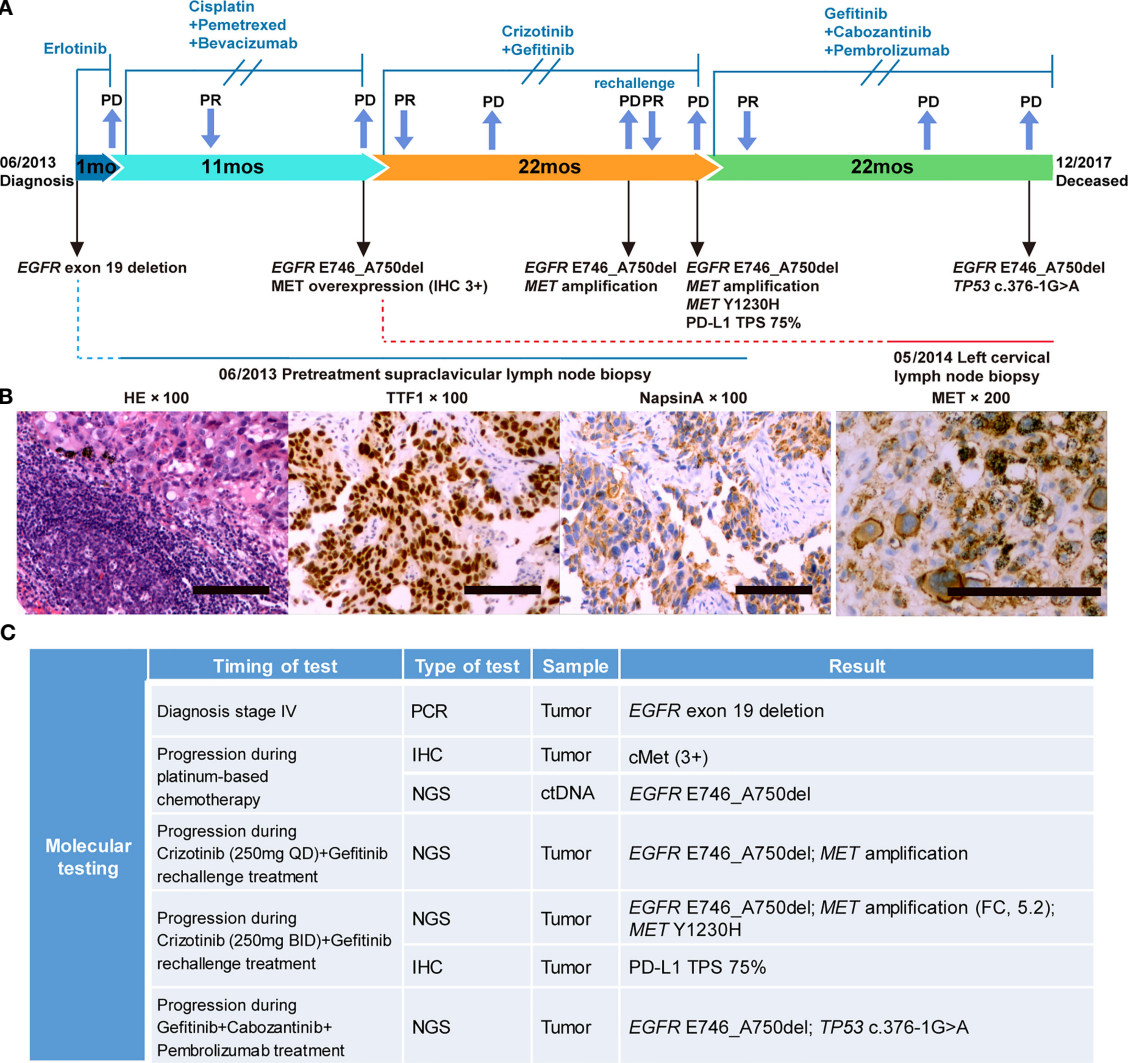
Case summary. **(A)** Summary of disease course, treatment timeline, and key molecular findings. **(B)** Hematoxylin-eosin staining, TTF1 and NapsinA positive immunohistochemical (IHC) staining before treatment; MET positive immunohistochemical staining during progression after platinum-based chemotherapy. Scale bars: 100 µm. **(C)** Detailed molecular alterations of tissue and liquid biopsy. FC, fold change; TPS, tumor proportion score.

A 49-year-old Chinese female never-smoker without personal or family history was diagnosed with stage IV NSCLC (T4N3M1b) in June 2013 (**Supplemental Table 1**). PCR testing of the biopsy revealed the presence of *EGFR* exon 19 deletion (ex19del) mutation. The treatment timeline and molecular alterations are shown in **Figure 1**. The patient was administered erlotinib (150 mg daily). Progressive disease in the right lower lobe of lung and supraclavicular lymph nodes was noted after one month. Treatment was then changed to chemotherapy with cisplatin, pemetrexed, and bevacizumab for 6 cycles with partial response followed by 5 cycles of pemetrexed and bevacizumab maintenance.

**Figure 2:**
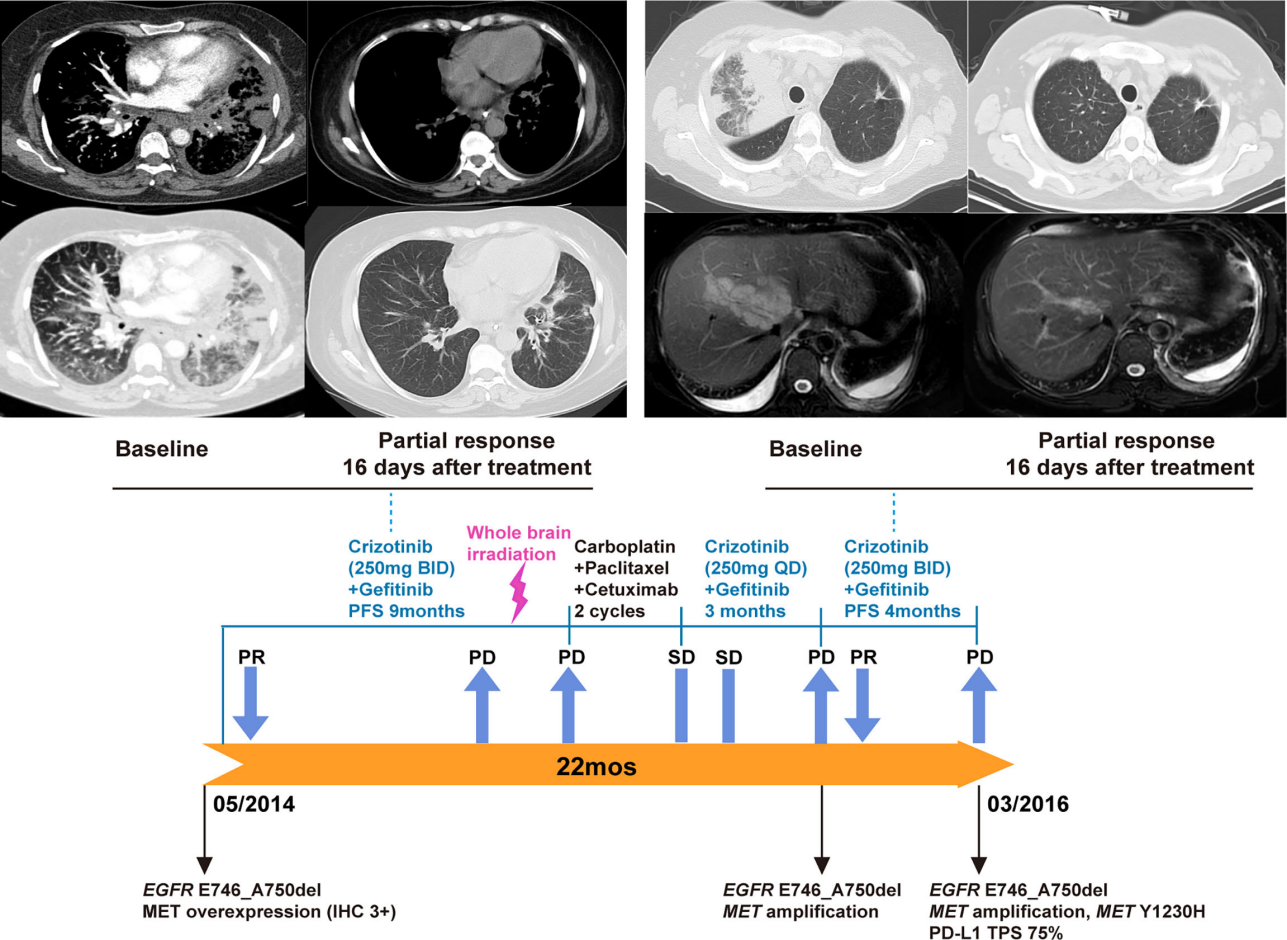
Gefitinib plus crizotinib treatment and rechallenge for *EGFR*-mutated NSCLC with *MET* overexpression/amplification. Based on the result of MET overexpression, the patient began gefitinib plus crizotinib. A partial response was observed 16 days after the initiation of this combination therapy (upper left panel). After disease progression, therapy was changed to chemotherapy plus cetuximab for two cycles. Due to chemotherapy-related toxicity, the patient was rechallenged with gefitinib plus crizotinib (reduced dose, 250 mg QD) with stable disease. Upon the development of new liver metastases, molecular testing of a biopsy confirmed concurrent *EGFR* mutation and *MET* amplification. Crizotinib dose was then increased to 250 mg BID (lower panel). Another partial response was observed 16 days after the crizotinib dose increase (upper right).

In May 2014, the patient reported neck swelling, upper limb edema, dyspnea, and dysphagia with a PS score of 4. The barium swallow study demonstrated the presence of esophageal stricture. Imaging revealed new bilateral pulmonary nodules and supraclavicular lymph nodes enlargement. Her oxygen saturation values dropped to 70-85%, and she received supplemental oxygen by noninvasive ventilation. A biopsy revealed MET overexpression (IHC 3+), and ctDNA next-generation sequencing confirmed the *EGFR* E746_A750del mutation (**Figures 1A, B**). Crizotinib is an ALK, ROS1, and MET tyrosine kinases inhibitor approved for advanced *ALK*-positive lung cancer at that time (16). Crizotinib has shown antitumor activity in lung cancer patients with *de novo MET* amplification (17, 18), and was under validation in clinical trials (NCT01441128, NCT00585195). Therefore, treatment was changed to crizotinib (250 mg BID) plus gefitinib (250 mg QOD). On day 5, the patient’s neck swelling, upper limb edema, dyspnea, and dysphagia improved. Her oxygen saturation values improved to 85-95%, and ventilation was discontinued. After 16 days, computed tomography imaging showed an almost complete reduction of the target lesions (**Figure 2**). In March 2015, disease progression occurred, and she underwent radiotherapy (DT40Gy/20F) for metastatic lesions of the brain. In May 2015, treatment was changed to a combination of carboplatin, paclitaxel, and cetuximab for two cycles, and discontinued due to grade 4 myelosuppression. The patient reported dyspnea and chest pain with a PS score of 3. She was rechallenged with gefitinib plus crizotinib (reduced dose, 250 mg QD) for three months with stable disease. Her dyspnea improved, but chest pain remained. In November, imaging revealed new liver metastases and progressive disease in the lung. Genomic profiling of a biopsy confirmed the same *EGFR* ex19del mutation and *MET* amplification. Crizotinib was increased to 250 mg BID. On day 16, imaging showed a dramatic improvement of the target lesions (**Figure 2**).

**Figure 3:**
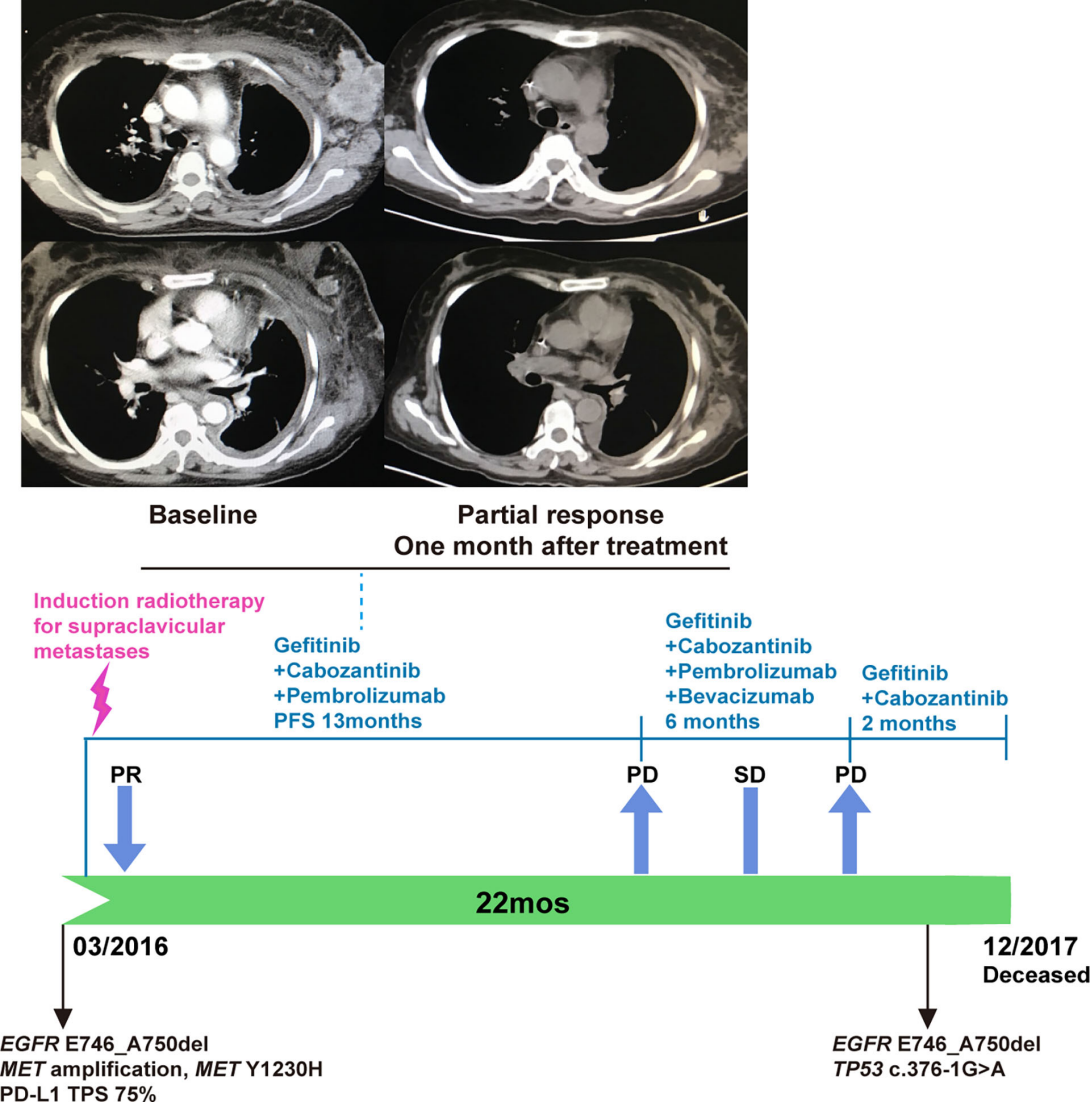
The combination of gefitinib, cabozantinib, and pembrolizumab for PD-L1-positive, *EGFR*-mutated, *MET*-amplified, *MET* Y1230H NSCLC. A partial response was observed one month after the initiation of this triplet regime (upper panel). Bevacizumab was added when the patient developed new metastases in April 2017. Both bevacizumab and pembrolizumab were stopped in October 2017 due to acute patient deterioration (lower panel).

In February 2016, the patient developed progressive dyspnea, cough, left-sided limb edema, chest pain, swelling of her left breast, and multiple chest wall/pulmonary nodules. Analysis of her left breast biopsy revealed an acquired *MET* Y1230H mutation and a high PD-L1 expression level (TPS 75%). She received induction radiotherapy (DT18Gy/3F) for supraclavicular metastases. The function of *MET* Y1230H mutation in lung cancer was unknown at that time. One *in vitro* study demonstrated that Y1230H mutation resulted in resistance to type I but not type II MET inhibitors in BaF3 cells (19). We reasoned that a type II MET inhibitor might overcome this acquired resistance. Additionally, given the latest approval of PD-1 antibody pembrolizumab in NSCLC and its distinct mechanism of action, we believed that pembrolizumab could benefit this patient independent of targeted therapy. With the informed consent from the patient, she received a salvage therapy comprising of gefitinib (250 mg QOD), cabozantinib (40 mg QD), and pembrolizumab (100 mg every two weeks). At one-month follow-up, her chest wall nodules and left breast swelling regressed, and her dyspnea improved. Imaging demonstrated a dramatic radiographic response which lasted for 13 months (**Figure 3**). In April 2017, the patient developed new metastases in the left erector spinae muscle and posterior abdominal wall. Bevacizumab was added to the combination regime with stable disease. Unfortunately, the patient’s condition further deteriorated in October. Cognitive deficits and electroencephalograms (EEG) abnormalities were noted. Imaging revealed pleural effusion and new lesions in the liver. Both bevacizumab and pembrolizumab were discontinued. Genomic profiling of a biopsy revealed the original *EGFR* mutation, a *TP53* c.376-1G>A splice site mutation, and the clearance of *MET* amplification/Y1230H mutation (**Figure 1**). The patient chose to continue gefitinib plus cabozantinib, and died of multiple organ dysfunction two months later. From the diagnosis of metastatic *EGFR*-mutant NSCLC in 2013, this patient achieved an overall survival of 54 months.

## Discussion

In the past decade, precision therapy has gradually become the standard of care for metastatic NSCLC patients with actionable biomarkers (20). These predictive biomarkers include immune biomarker PD-L1 and targetable driver mutations such as *EGFR* L858R/ex19del mutations. While next-generation sequencing and PD-L1 testing have significantly improved the treatment decision-making process for NSCLC, oncologists need to realize that the optimal treatment for patients with multiple actionable biomarkers requires evidence-based biological rationale and up-to-date knowledge of clinical trial results.

The *EGFR*-mutant NSCLC case we reported involved the interplay of four biomarkers: *EGFR* exon 19 deletion (ex19del), PD-L1, *MET* amplification (*MET*-amp), and *MET* Y1230H mutation. To make the discussion more relevant to today’s clinical practice, we constructed three scenarios related to this case: 1. *EGFR* ex19del mutation and high PD-L1 expression in untreated NSCLC patients; 2. acquired *MET* amplification in *EGFR* ex19del NSCLC after first-line EGFR TKI therapy; 3. acquired *MET* Y1230H mutation in *MET*-driven NSCLC after first-line MET TKI therapy.

For the first scenario, *EGFR* ex19del NSCLC patients with high PD-L1 expression levels, three treatment choices are available: chemotherapy, EGFR-targeted therapy, and immunotherapy. The decision-making process for this scenario can be simplified by a review of NSCLC NCCN guidelines and literature. The 2017 NSCLC NCCN guideline stated that EGFR TKIs resulted in longer PFS and fewer toxicities than chemotherapy in patients with sensitizing *EGFR* mutations (21). Additionally, pembrolizumab did not show any response in the first 11 patients enrolled in a phase 2 trial for *EGFR*-mutant, PD-L1-positive NSCLC, including 8 patients with PD-L1 expression more than 50% (22). In a multicenter, retrospective study involving 171 *EGFR*-mutant NSCLC patients treated with immunotherapy, subgroup analysis demonstrated that the response rate of PD(L)-1 antibodies was very low in *EGFR* ex19del NSCLC irrespective of the PD-L1 status (23). Furthermore, the combination of EGFR TKIs and immunotherapy had significantly higher toxicities than either alone (24). For these reasons, Calles et al. commented in the 2020 ASCO educational book that immunotherapy alone or combined with EGFR TKI is not recommended to *EGFR*-mutant NSCLC (25). For the first scenario, EGFR TKI should be the first-line therapy choice.

The second scenario, EGFR TKI resistance mediated by *MET* amplification, is a frontier currently under intensive investigation (26). There are at least three possible strategies to overcome this challenge: EGFR TKI plus MET TKI, EGFR-MET bispecific antibodies, and EGFR TKI plus EGFR-MET bispecific antibodies. The EGFR/MET TKI combinations evaluated in early-stage clinical trials include osimertinib/savolitinib, gefitinib/savolitinib, gefitinib/capmatinib, and gefitinib/tepotinib. In the phase 1b TATTON study, osimertinib plus savolitinib achieved an ORR of 64% in osimertinib-naive, *EGFR* T790M-negative patients and 48% in a mixed pool of osimertinib-treated, osimertinib-naive/T790M-positive, and osimertinib-naive/T790M-negative patients (15). A phase 1b trial of gefitinib plus savolitinib showed an ORR of 52% (12/23), 9% (2/23), and 40% (2/5) in *EGFR* T790M-negative, -positive, and -unknown patients, respectively (12). In a phase 1b/2 trial, gefitinib plus capmatinib reached an ORR of 47% in patients with high *MET*-amplification (*MET* gene copy number ≥ 6) and 27% overall (14). Similarly, in an early-terminated phase 2 trial (INSIGHT), subgroup analysis revealed that gefitinib plus tepotinib resulted in longer PFS/OS than platinum duplet chemotherapy control in 34 patients with high MET overexpression (IHC3+) (mPFS 8.3 vs 4.4 months, HR 0.35; mOS 37.3 vs 17.9 months, HR 0.33) as well as 19 patients with high MET amplification (mean gene copy number ≥5 or MET to the centromere of chromosome 7 ratio ≥2) (mPFS 16.6 vs 4.2 months, HR 0.13; mOS 37.3 vs 13.1 months, HR 0.08) (13). Our patient had high MET overexpression (IHC 3+) and high *MET* amplification (*MET* gene copy number = 5.2). Although she received different gefitinib/MET TKI combination therapies (gefitinib/crizotinib and gefitinib/cabozantinib), her PFS and OS are similar to the results of the INSIGHT study (gefitinib/tepotinib).

In May 2021, FDA granted the accelerated approval of an EGFR-MET bispecific antibody amivantamab as the first targeted therapy for NSCLC patients with *EGFR* exon 20 insertions (ex20ins) based on the phase 1 CHRYSALIS study (27). Amivantamab inhibits EGFR/MET signaling through three mechanisms: internalization and degradation of EGFR/MET receptors, blocking ligand-dependent receptor activation, and antibody-dependent cell-mediated cytotoxicity (ADCC) (28). In a preclinical model of *EGFR*-mutant, *MET*-amplified NSCLC, amivantamab showed superior antitumor activity than the combination of erlotinib and crizotinib (29). Interestingly, results of the CHRYSALIS trial demonstrated that amivantamab alone or combined with a third-generation EGFR TKI lazertinib had antitumor activity in patients with *EGFR*-mutant, *MET*-amplified NSCLC (30). The subgroup analysis showed that amivantamab plus lazertinib had a 90% (9/10) and 10% (1/10) response rate in MET IHC-high and MET IHC-low patients, respectively (31). Therefore, amivantamab could be the game-changer for this specific patient population.

The third scenario, MET TKI resistance mediated by *MET* Y1230H mutation, is associated with *MET*ex14 and *MET*-amplified NSCLC. Currently, the approval of capmatinib, tepotinib, and salvotinib are limited to *MET*ex14, which is the only *MET* alteration included in the 2021 NSCLC NCCN guideline (2). According to the TCGA data, *MET*ex14 and *MET* amplification were the first and second most frequent *MET* alterations in NSCLC, respectively (32). In agreement, Y1230H mutation was more frequently observed in *MET*ex14 NSCLC than *MET*-amplified NSCLC (33, 34). Works from Engstrom and others confirmed that MET Y1230H mutant was resistant to type I MET TKI capmatinib, salvotinib, and crizotinib but remained sensitive to type II MET TKI glesatinib (10). As type I and II MET TKIs bind the ATP-pocket of MET differently, they have distinct inhibition capacities to MET mutants. The IC50 values of *MET*ex14 single mutant for capmatinib, crizotinib, and glesatinib in NIH/3T3 cells were 2.4 nM, 28.9 nM, and 80.6 nM, respectively. In contrast, the IC50 values of *MET*ex14/Y1230H double mutant for capmatinib, crizotinib, and glesatinib in NIH/3T3 cells were >3,000 nM, 278 nM, and 56 nM, respectively (10). Similarly, the IC50 values of MET Y1230H single mutant for capmatinib, savolitinib, crizotinib, glesatinib, cabozantinib, merestinib in Ba/F3 cells were 401 nM, >1,000 nM, 216 nM, 19 nM, 20 nM, and 8.2 nM, respectively (35). These results predict that acquired *MET* Y1230H mutation will confer resistance to all MET TKIs currently approved for NSCLC.

Given the fact that cabozantinib is the only approved type II MET inhibitor, the combination of cabozantinib and EGFR TKI is a reasonable and feasible strategy to combat acquired resistance to type I MET TKI. Bahcall et al. reported acquired D1228V mutation resulted in resistance to osimertinib plus salvotinib in an *EGFR* ex19del/*MET*-amp NSCLC patient, who subsequently responded to erlotinib plus cabozantinib (36). In another similar case, plasma genotyping revealed four acquired *MET* mutations (D1228H/N/Y and Y1230C) in an *EGFR* ex19del/*MET*-amp NSCLC patient who became resistant to osimertinib plus salvotinib (37). Treatment was changed to osimertinib plus cabozantinib with stable disease and clearance of Y1230C mutation. However, cabozantinib was soon discontinued due to toxicities, and the patient died three months later. These clinical observations are consistent with the report that *MET* D1228X mutations are more resistant to cabozantinib than Y1230X mutations (38).

Lastly, we would like to discuss our treatment decision-making for crizotinib resistance mediated by *MET* Y1230H mutation. In February 2016, we could not find reports of this mutation in lung cancer except for two preclinical MET TKI resistance studies (19, 39). Tiedt et al. conducted a drug resistance screen in BaF3 TPR-MET cells with type I MET inhibitor NVP-BVU972 and type II MET inhibitor AMG-458 (19). Most NVP-BVU972-resistant clones carry missense mutations in Y1230 and D1228. Structure study revealed that NVP-BVU972 interacts with the aromatic side chain of Y1230. Therefore, a mutation in this residue will disrupt NVP-BVU972 binding and result in drug resistance. The Y1230 mutation was not detected in the AMG-458 screen. Biochemical assay results demonstrated that MET Y1230H mutant was sensitive to AMG-458 but not NVP-BVU972 (IC50 value: 1.6 nM vs. >127 nM). Similarly, Funakoshi et al. conducted a screen in a *MET*-amplified gastric cell line MKN45 with a type I MET inhibitor PHA665752 and a type II MET inhibitor GSK1363089/XL880/foretinib (39). *MET* Y1230H mutation was only identified in PHA665752-resistant clones but not foretinib-resistant clones. This result was expected as the IC50 value of Y1230H mutant for XL880/foretinib was only 0.7 nM (19). Based on these results, we reasoned that the crizotinib resistance seen in our case was likely mediated by Y1230H, a *MET* mutation sensitive to type II MET inhibitors. Therefore, we replaced type I MET TKI crizotinib with type II MET TKI cabozantinib in our EGFR/MET dual blockade regime.

In summary, we presented the efficacy of gefitinib plus crizotinib in an *EGFR*-mutant NSCLC patient with high-level *MET* overexpression/amplification and resistance to erlotinib. Because crizotinib is more accessible and affordable than capmatinib, tepotinib, and salvotinib, this combination provides a feasible treatment option for *EGFR*-mutant, *MET*-amplified NSCLC patients who can not assess or afford MET-specific TKIs. Furthermore, the switch from type I MET TKI to type II MET TKI cabozantinib can be an effective strategy to overcome acquired type I MET TKI resistance in NSCLC. Given the recent approval of EGFR-MET bispecific antibody amivantamab, future investigations are required to explore the safety and efficacy of TKI-based and antibody-based EGFR/MET dual blockade therapy in *EGFR*-mutant, *MET*-amplified NSCLC.

## Data Availability Statement

The original contributions presented in the study are included in the article/**Supplementary Material**. Further inquiries can be directed to the corresponding authors.

## Ethics Statement

Written informed consent was obtained from the individual(s) for the publication of any potentially identifiable images or data included in this article.

## Author Contributions

FL: conceptualization, methodology, supervision, and writing-reviewing and editing. BC and XL: visualization, investigation, and writing-original draft preparation. XH, BQ, WYu, WYang, PZ, and JC: investigation, data curation, and validation. TM and BQ: writing-reviewing and editing. All authors contributed to the article and approved the submitted version.

## Conflict of Interest

XL and TM are employees of Genetron Health (Beijing) Technology, Co. Ltd.

The remaining authors declare that the research was conducted in the absence of any commercial or financial relationships that could be construed as a potential conflict of interest.

## Publisher’s Note

All claims expressed in this article are solely those of the authors and do not necessarily represent those of their affiliated organizations, or those of the publisher, the editors and the reviewers. Any product that may be evaluated in this article, or claim that may be made by its manufacturer, is not guaranteed or endorsed by the publisher.
