# Supplementary material for: Case Report: Sequential Combination Targeted Therapy With Type I and II MET Inhibitors in a Metastatic EGFR-Mutated, MET-Amplified NSCLC Patient With Acquired MET Y1230H Mutation
Source: Front Oncol. 2021 Dec 2;11:738832. doi: 10.3389/fonc.2021.738832 (PMC8674488; doi:10.3389/fonc.2021.738832)
Supplement: Supplementary file 1 [file Table_1.pdf]

Supplemental Table 1. Demographic and clinical characteristics of the patient at diagnosis.

Characteristic	
Age	49
Gender	Female
Race	Asian (Chinese)
Smoking history	No
Histology type	Adenocarcinoma
PS score	1
Clinical stage at diagnosis	IV (cT4N3M1b)
Coexisting conditions	Hypertension for more than 20 years
Family history of malignancy	No
EGFR mutation type	*EGFR* exon 19 deletion

PS, performance status.
